# Are ultra-processed foods really cheap? Reassessing health, equity and environmental costs across the life course in low- and middle-income countries

**DOI:** 10.1017/S1368980026102778

**Published:** 2026-05-22

**Authors:** Sandeep Kaur, Akashdeep Singh Chauhan

**Affiliations:** 1 Surgery and Cancer, Imperial College London, UK; 2 Indian Institute of Public Health-Delhi, Public Health Foundation of India, India

**Keywords:** Ultra-processed foods, NOVA classification, Non-communicable diseases, School-based interventions, Food policy, Food marketing

## Abstract

Ultra-processed foods, as classified by NOVA, provide a substantial and rising share of dietary energy worldwide. Manufactured from cheap refined ingredients and cosmetic additives, they are engineered for hyper-palatability, long shelf life and profit and are widely perceived as convenient, low-cost options. This commentary argues that such perceptions of ‘cheapness’ are misleading once health, equity and environmental impacts are considered.

Ultra-processed foods (UPF), as defined by NOVA, have become central to contemporary diets, particularly in high-income countries and increasingly in low- and middle-income settings^(1)^. These industrial formulations, based on refined flours, sugars and oils, protein isolates and starches, plus cosmetic additives, are engineered for hyper-palatability, shelf life and brand differentiation rather than nutritional value^(2)^. They are marketed aggressively, including to children, and are widely perceived as affordable, convenient staples. Across low- and middle-income countries, household food baskets are shifting away from traditional minimally processed staples towards UPF^(2)^. The apparent ‘cheapness’ of UPF is largely confined to their low shelf price per kilocalorie. When long-term health impacts, social consequences and environmental externalities are taken into account, UPF emerge as costly to individuals, health systems and ecosystems^(3)^.

## Ultra-processed foods and the illusion of affordability

UPF are designed to transform low-value agricultural commodities into high-profit products. They rely on refined flours, sugars and vegetable oils, combined with protein isolates, modified starches, maltodextrins, hydrogenated or interesterified fats and a wide range of cosmetic additives, using processes such as extrusion, moulding and pre-frying^(3)^. Economies of scale and long shelf life reduce waste and retail risk, underpinning low unit prices, particularly when cost is expressed per kilocalorie. Unsurprisingly, lower-income households in multiple countries obtain a disproportionate share of their energy from UPF^(4)^. However, such affordability calculations ignore long-term health-care costs, reduced productivity, cultural disruption and environmental damage.

Dietary surveys consistently show that replacing minimally processed foods and basic culinary ingredients with UPF leads to more energy-dense diets, higher intakes of free sugars and saturated fats and lower intakes of fibre and key micronutrients^(5)^. In Brazil, increasing purchases of ready-to-eat and ready-to-heat UPF have coincided with declining purchases of rice, beans, roots and tubers, vegetables and traditional culinary oils^(3)^. Similar patterns have been documented elsewhere, with higher UPF intake predicting poorer overall diet quality^(5,6)^. Beyond nutrients, this displacement erodes culinary skills, weakens family meals and reduces cultural and dietary diversity. In this light, the low retail price of UPF represents a shifting of costs from manufacturers and retailers onto households and public systems^(3)^.

## Epidemiological evidence and mechanisms

A large and rapidly expanding literature links UPF consumption to adverse health outcomes^(5)^. Systematic reviews report that higher UPF intake is consistently associated with increased prevalence of overweight and obesity, larger waist circumference, abdominal obesity and metabolic syndrome and with lower HDL-cholesterol^(7,8)^. Studies from the high-income countries show that greater UPF consumption predicts incident overweight/obesity, hypertension, frailty, cardiovascular events, overall and breast cancer, depression and elevated all-cause and cardiovascular mortality^(5,9)^. Across settings, the direction of association is consistently adverse, and dose–response gradients are frequently observed^(9,10)^.

Multiple mechanisms plausibly explain these findings. UPF are energy dense and high in rapidly digestible carbohydrates, driving high glycaemic loads and promoting excess energy intake and weight gain^(5,8)^. They are low in fibre and micronutrients, undermining satiety, glycaemic control and cardiometabolic regulation^(10)^. Their altered structures and textures often facilitate rapid ingestion and reduced Oro-sensory exposure, weakening satiety signalling and favouring larger portion sizes^(8)^. Processing and packaging introduce additional exposures, including contaminants such as acrylamide and chemicals such as bisphenols and phthalates, which have been linked to cardiometabolic and endocrine disruption^(11)^. Critically, UPF displace minimally processed, protective foods, diminishing intake of cardioprotective nutrients and bioactive compounds and undermining dietary patterns known to prevent non-communicable diseases (NCD)^(5,12)^. Together, this evidence supports recognition of ultra-processing itself as an independent dimension of dietary risk, not captured by nutrient profiles alone^(13)^.

## Life-course and equity perspectives

A life-course lens underscores the importance of UPF exposure in childhood and adolescence. Young people consume large and increasing quantities of sugar-sweetened beverages, confectionery, packaged snacks and ready-to-eat meals, particularly in rapidly transitioning food environments^(4,7,14)^. Chile and other Latin American countries growth in sales of ready-to-eat meals and snack products has paralleled rising youth obesity^(4,7)^. Dietary habits and body-weight trajectories established in childhood tend to persist, and higher UPF intake in youth predicts later weight gain and metabolic risk^(8)^. Given strong links between childhood obesity and adult cardiovascular diseases (CVD), diabetes and some cancers, UPF-rich diets in early life likely contribute substantially to adult NCD burdens^(12)^. Because NCD are chronic and costly to manage, early exposure transforms short-term savings at the checkout into long-term clinical and economic liabilities for families and health systems^(14)^.

Equity is central to this discussion. UPF consumption is socially patterned, with low-income and marginalised groups often relying more heavily on such products due to lower prices per kilocalorie, long shelf life, dense retail availability and targeted marketing, alongside limited access to affordable minimally processed foods^(6,11)^. As a result, the health and environmental harms associated with UPF risk disproportionately affecting those with the fewest resources, widening existing inequalities in NCD and environmental exposures^(12,15)^. Any policy response must therefore be designed explicitly to reduce, rather than exacerbate, socio-economic gradients in diet-related health.

## Environmental and commercial determinants

UPF also exert substantial environmental and social impacts that are not reflected in their retail price^(8)^. They rely on refined commodities such as sugar and vegetable oils, often linked to deforestation, biodiversity loss and greenhouse gas emissions, and require energy-intensive processing, refrigeration, storage and transport^(11)^. Extensive plastic and composite packaging contributes to solid waste and chemical pollution, including endocrine-disrupting substances^(11)^. Because many UPF are discretionary and not required for adequate nutrition, their environmental footprint is largely avoidable; shifting consumption towards minimally processed plant-based foods offers clear co-benefits for health and climate^(11)^.

The proliferation of UPF is driven by powerful commercial actors. Transnational food and beverage corporations deploy aggressive marketing and branding, including child-targeted advertising, sponsorship and product placement, to embed UPF in daily routines and retail environments^(16)^. These practices normalise UPF as everyday staples and frame diet as a matter of individual responsibility, obscuring the structural and corporate determinants of food environments. In effect, the low shelf price of UPF is achieved by externalising substantial health and environmental costs to public systems and future generations^(11,16)^.

## Schools as a strategic setting

Schools are a particularly strategic setting for addressing UPF consumption. In this commentary, we focus primarily on low-and-middle income countries (LMIC), where school food environments often remain highly permissive to UPF^(14,15)^. Children and adolescents spend much of their day in or around schools, where food choices are shaped by canteen offerings, vending machines, nearby retailers and peer norms and where marketing and branding are often visible. Evidence indicates that UPF are deeply embedded in school food environments, displacing home-prepared meals and traditional snacks and reinforcing habits of frequent snacking and sugary drink consumption^(14,15,17)^. This picture contrasts with several high-income countries, where nutritional standards and regulations now severely restrict many UPF in school meals and vending. Because school age is a critical period for habit formation, school-based interventions can have durable impacts on dietary trajectories^(14,15,17)^.

Cluster-randomised trials in India and Brazil show that school-based behavioural interventions and parental education can reduce UPF and added sugar intake and improve diet quality^(14,15,17)^. Effective policy packages typically include bans or strict limits on sugar-sweetened beverages, packaged snacks and confectionery on school premises; nutrition standards for school meals that emphasise minimally processed foods and curriculum-based education on food processing, label reading and marketing literacy^(14,15,17)^. Locating such measures within a broader structural approach, rather than relying on individual advice alone, is consistent with evidence on the commercial determinants of UPF consumption^(14–17)^.

## Policy implications

The converging evidence on health, environmental and social harms implies that treating UPF as acceptable low-cost staples is no longer tenable. Structural policies are needed to make their hidden costs visible and to rebalance food environments^(11)^. Most concrete policy experience to date comes from upper-middle-income countries (notably Chile and Mexico), with more limited implementation so far in low-income settings. High-income countries have also adopted some measures, but the mix and strength of policies vary substantially^(18,19)^. Chile, Mexico and other Latin American countries have introduced black octagonal ‘high in’ warnings for sugar, salt, saturated fat and energy, backed by nutrient profile models^(18–20)^. Prior experience from these countries suggests that nutrient-based warnings can reduce purchases of heavily flagged products and stimulate reformulation. Interpretive front-of-pack nutrition labelling, using nutrient warnings or traffic-light systems, can further help consumers rapidly identify products high in free sugars, saturated fats, Na or energy and partially counter marketing cues^(18,19)^. Given the specific concern about processing, there is a strong rationale for complementing nutrient-based labels with indicators of processing level, addressing well-documented difficulties in recognising UPF from ingredient lists alone^(20)^.

Marketing restrictions, especially to protect children, represent a second priority. Policies can include banning or severely limiting advertising of UPF high in sugar, salt and saturated fat during children’s programming and on digital platforms heavily used by children, prohibiting child-appealing characters, toys and promotions on packaging and removing brand promotion from schools^(18–20)^. Evidence from restrictions on sugar-sweetened beverages and fast food indicates that such measures can reduce exposure and may shift preferences and purchasing patterns. However, many low- and middle-income countries still have minimal or poorly enforced restrictions on UPF marketing, particularly in digital spaces, and there is a paucity of rigorous evaluations from these settings.

Fiscal measures can realign prices with health objectives. Taxes on sugar-sweetened beverages have reduced purchases and prompted reformulation in several jurisdictions; similar approaches could be extended to other UPF high in free sugars or saturated fats, especially if revenues are earmarked for subsidies on fruits, vegetables and minimally processed staples^(2,6)^. To avoid regressive effects, taxes should be paired with targeted subsidies and social protection to ensure that healthier options are accessible and affordable, particularly for low-income households^(6,8)^.

Finally, environmental and sustainability policies should explicitly incorporate processing, packaging and distribution. Recognising UPF as contributors to greenhouse gas emissions, resource use and waste justifies their inclusion in dietary guidelines and climate strategies^(11)^. Measures such as extended producer responsibility and restrictions on single-use plastics are likely to disproportionately affect UPF-dominated sectors and can encourage less resource-intensive models of food production and distribution^(11)^.

## Strengths, limitations and research needs

Evidence on UPF and health is remarkably consistent and broad, spanning multiple continents, populations and outcomes. However, most data are observational, leaving room for residual confounding and measurement error. Dietary misreporting, variability in NOVA classification and heterogeneity within UPF categories complicate interpretation^(20)^. Mechanistic understanding of specific additives, processing methods and packaging-related exposures remains incomplete, and robust evaluations of UPF-focused policies are still emerging^(18,19)^. Future work should refine UPF definitions, improve exposure assessment, deepen mechanistic insight and evaluate the effectiveness and equity impacts of structural policies.

## Conclusion

UPF now provide a large and growing share of dietary energy and are widely perceived as convenient, low-cost foods. Yet convergent evidence shows that high UPF consumption is associated with poorer diet quality, greater adiposity, metabolic disturbances and higher risks of major NCD and premature mortality, and that it contributes to environmental degradation and social inequities^(5,11)^. In many low- and middle-income countries, household food baskets are shifting away from traditional minimally processed staples towards UPF, often in the absence of strong regulatory protections in schools and other settings^(15)^. The emerging experiences of countries, such as Chile and Mexico demonstrate that structural policies, front-of-pack warning labels, marketing restrictions and taxes on sugar-sweetened beverages, can help to curb consumption of unhealthy products, but comprehensive UPF-focused policy packages remain rare and under-evaluated, particularly in low-income and lower-middle-income contexts^(18,19)^. UPF are thus only superficially cheap: they are inexpensive at the checkout but costly over the life course and across society. Applying the precautionary principle, there is a strong case for decisive structural action, through labelling, marketing controls, fiscal policies, environmental regulation and school-centred interventions, to reduce reliance on UPF and realign food systems with the long-term health of people, and the planet will require policies that make minimally processed, nutrient-dense foods the truly affordable option.

## References

[1] Gibney MJ (2018) Ultra-processed foods: definitions and policy issues. Curr Dev Nutr 3, nzy077. doi:10.1093/cdn/nzy077. 30820487 PMC6389637

[2] Pagliai G , Dinu M , Madarena MP et al. (2021) Consumption of ultra-processed foods and health status: a systematic review and meta-analysis. Br J Nutr 125, 308–318. doi:10.1017/S0007114520002688. 32792031 PMC7844609

[3] Monteiro CA , Levy RB , Claro RM et al. (2011) Increasing consumption of ultra-processed foods and likely impact on human health: evidence from Brazil. Public Health Nutr 14, 5–13. doi:10.1017/S1368980010003241. 21211100

[4] Marino M , Puppo F , Del Bo’ C et al. (2021) A systematic review of worldwide consumption of ultra-processed foods: findings and criticisms. Nutrients 13, 2778. doi:10.3390/nu13082778. 34444936 PMC8398521

[5] Srour B , Kordahi MC , Bonazzi E et al. (2022) Ultra-processed foods and human health: from epidemiological evidence to mechanistic insights. Lancet Gastroenterol Hepatol 7, 1128–1140. doi:10.1016/S2468-1253(22)00169-8. 35952706

[6] Mialon M & Gomes FDS (2019) Public health and the ultra-processed food and drink products industry: corporate political activity of major transnationals in Latin America and the Caribbean. Public Health Nutr 22, 1898–1908. doi:10.1017/S1368980019000417. 30859929 PMC10260506

[7] Barquera S , Hernandez-Barrera L , Tolentino ML et al. (2008) Energy intake from beverages is increasing among Mexican adolescents and adults. J Nutr 138, 2454–2461. doi:10.3945/jn.108.092163. 19022972

[8] Lane MM , Gamage E , Du S et al. (2024) Ultra-processed food exposure and adverse health outcomes: umbrella review of epidemiological meta-analyses. BMJ 384, e077310. doi:10.1136/bmj-2023-077310. 38418082 PMC10899807

[9] Canhada SL , Luft VC , Giatti L et al. (2020) Ultra-processed foods, incident overweight and obesity, and longitudinal changes in weight and waist circumference: the Brazilian Longitudinal Study of Adult Health (ELSA-Brasil). Public Health Nutr 23, 1076–1086. doi:10.1017/S1368980019002854. 31619309 PMC7282862

[10] Martini D , Godos J , Bonaccio M et al. (2021) Ultra-processed foods and nutritional dietary profile: a meta-analysis of nationally representative samples. Nutrients 13, 3390. doi:10.3390/nu13103390. 34684391 PMC8538030

[11] Seferidi P , Scrinis G , Huybrechts I et al. (2020) The neglected environmental impacts of ultra-processed foods. Lancet Planet Health 4, e437–e438. doi:10.1016/S2542-5196(20)30177-7. 33038314

[12] Chen X , Chu J , Hu W et al. (2022) Associations of ultra-processed food consumption with cardiovascular disease and all-cause mortality: UK Biobank. Eur J Public Health 32, 779–785. doi:10.1093/eurpub/ckac104. 36006020 PMC9527958

[13] Monteiro CA , Martínez-Steele E & Cannon G (2024) Reasons to avoid ultra-processed foods. BMJ 384, q439. doi:10.1136/bmj.q439. 38418096

[14] Kaur S , Kumar R & Kaur M (2026) School-based behaviour change intervention to reduce ultra-processed food consumption among adolescents: evidence from a cluster-randomised controlled trial in India. BMJ Glob Health 11, e020799. doi:10.1136/bmjgh-2025-020799. PMC12815165 41513306

[15] Kaur S , Kumar R , Lakshmi PVM et al. (2024) Effectiveness of a school-based behavioural change intervention in reducing chronic disease risk factors in Chandigarh, India: a cluster-randomised controlled trial. Lancet Reg Health Southeast Asia 21, 100353. doi:10.1016/j.lansea.2024.100353. 38312946 PMC10832458

[16] van Tulleken C (2025) Ultra-processed foods and public health: evidence of harm and of conflicts of interest in the food industry to evade regulation. Future Healthc J 12, 100263. doi:10.1016/j.fhj.2025.100263. 40692632 PMC12277474

[17] Baratto PS , Hoffman DJ , Valmórbida JL et al. (2025) Effectiveness of an intervention to prevent ultra-processed foods and added sugar in the first year of life: a multicentre randomised controlled trial in Brazil. J Hum Nutr Diet 38, e70022. doi:10.1111/jhn.70022. 39957417 PMC11831244

[18] D’Angelo Campos A , Ng SW , McNeel K et al. (2024) How promising are ‘Ultraprocessed’ front-of-package labels? A formative study with US adults. Nutrients 16, 1072. doi:10.3390/nu16071072. 38613105 PMC11013171

[19] Corvalán C , Reyes M , Garmendia ML et al. (2019) Structural responses to the obesity and non-communicable diseases epidemic: update on the Chilean law of food labelling and advertising. Obes Rev 20, 367–374. doi: 10.1111/obr.12802. 30549191

[20] Monteiro CA , Cannon G , Moubarac JC et al. (2018) The UN Decade of Nutrition, the NOVA food classification and the trouble with ultra-processing. Public Health Nutr 21, 5–17. doi:10.1017/S1368980017000234. 28322183 PMC10261019

